# Factors Associated with Lung Function Decline in Patients with Non-Tuberculous Mycobacterial Pulmonary Disease

**DOI:** 10.1371/journal.pone.0058214

**Published:** 2013-03-06

**Authors:** Meng-Rui Lee, Ching-Yao Yang, Kai-Ping Chang, Li-Ta Keng, David Hung-Tsang Yen, Jann-Yuan Wang, Huey-Dong Wu, Li-Na Lee, Chong-Jen Yu

**Affiliations:** 1 Department of Internal Medicine, National Taiwan University Hospital, Taipei, Taiwan; 2 Department of Internal Medicine, Taoyuan General Hospital, Taoyuan City, Taiwan; 3 Department of Internal Medicine, Kinmen Hospital, Kinmen, Taiwan; 4 Department of Emergency Medicine, Taipei Veterans General Hospital, National Yang-Ming University, Taipei, Taiwan; 5 Department of Laboratory Medicine, National Taiwan University Hospital, Taipei, Taiwan; National Institute of Infectious Diseases, Japan

## Abstract

**Background:**

There is paucity of risk factors on lung function decline among patients with non-tuberculous mycobacteria (NTM) pulmonary disease in literature.

**Methods:**

Patients with NTM pulmonary disease between January 2000 and April 2011 were retrospectively selected. Sixty-eight patients had at least two pulmonary function tests within a mean follow-up period of 47 months.

**Results:**

Sixty-eight patients were included. They had a median age of 65 years and 65% had impaired lung function (Forced expiratory volume in 1 second [FEV_1_] <80% of predicted value). The mean FEV_1_ decline was 48 ml/year. By linear regression, younger age (beta: 0.472, *p*<0.001), initial FEV_1_>50% of predicted value (beta: 0.349, *p* = 0.002), male sex (beta: 0.295, *p* = 0.018), bronchiectasis pattern (beta: 0.232, *p* = 0.035), and radiographic score >3 (beta: 0.217, *p* = 0.049) were associated with greater FEV_1_ decline. Initial FEV_1_>50% of predicted value (beta: 0.263, *p* = 0.032) was also associated with greater FVC annual decline, whereas *M. kansasii* pulmonary disease was marginally associated with greater annual FVC decline (beta: 0.227, *p* = 0.062).

**Conclusions:**

NTM pulmonary disease is associated with greater decline in lung function in patients who are young, male, with bronchiectasis, and with a high radiographic score. Special attention should be given to patients with these risk factors.

## Introduction

Pulmonary disease due to non-tuberculous mycobacteria (NTM) is an emerging problem worldwide due to its increasing incidence [1]. Because of its less rapidly progressive disease course, it is prone to be neglect in clinical practice [1]. Many NTM specimens, notably *Mycobacterium abscessus* complex, are multi-drug resistant to currently recommended treatment regimens [1]. Existing guidelines suggest treatment with multiple drug regimens for at least one year, with culture conversion as the indicator of treatment success [1]. The decision to treat NTM pulmonary disease is therefore difficult to make due to drug resistance and lengthy treatment course [1], [2]. It is therefore important to identify patients at greater risk for clinical deterioration and provide optimal therapy to those patients.

Pulmonary function test is a useful tool for assessing patient health status and as an indicator of adverse outcome in respiratory diseases [3]. Lung function has been correlated with quality of life, with most studies emphasizing its role in patients with chronic obstructive pulmonary disease [4]. In pulmonary tuberculosis (PTB) patients, lung function impairment and further decline after completing anti-tuberculosis treatment is well-documented [5]. In pulmonary NTM patients, there is also evidence that the presence of NTM species in the lungs signifies poorer baseline lung function [6], [7]. In one recent study, lung function is significantly associated with health-related quality of life (HRQL) in pulmonary NTM patients [8]. Despite the limited case number, treatment for NTM species reportedly improves pulmonary function in *Mycobacterium avium complex* (MAC) lung disease patients [9].

Few studies, however, have focused on pulmonary function change in patients with NTM pulmonary disease. This study aimed to identify risk factors of lung function decline among pulmonary NTM patients.

## Materials and Methods

### Ethics Statement

The Institutional Review Board of National Taiwan University Hospital approved the study (NTUH REC: 9561707008). The Institutional Review Board waved the need for informed consent because this retrospective study used an encrypted database and did not add any risk to the participants.

### Subjects

This study was conducted at the National Taiwan University Hospital, a 2900-bed tertiary-care centre in northern Taiwan. The mycobacterial laboratory registry database covering the period January 2000 to April 2011 was reviewed to identify patients with at least two respiratory specimens that were culture-positive for non-tuberculous mycobacteria. Patients with at least two pulmonary function tests during the follow-up or treatment period were further selected. The clinical significance of NTM isolates and the adequacy of treatment were judged according to the ATS guidelines [1], wherein patients were considered as having NTM pulmonary disease if they fulfilled the following criteria: (1) at least two sputum or one bronchial washing/brushing sample, or one lung tissue culture-positive for the same NTM species; (2) presence of respiratory symptoms; (3) chest radiography or computed tomography (CT) demonstrating new patch(es) of consolidation, exudative, nodular infiltrates, cavitary lesions, or multi-focal bronchiectasis; and (4) exclusion of other pulmonary causes [1], [10]. Only those with NTM pulmonary disease were included.

All respiratory specimens sent for mycobacterial culture were processed as previously described [11]. Briefly, NaOH-citrate–N-acetyl-L-cysteine was added to each specimen in an equal volume and allowed to settle at room temperature for 15 min. After centrifugation, the precipitate was re-suspended in 1.5 ml phosphate-buffered saline (pH 7.4). Culture was performed by inoculating 0.5 ml of sediment onto a Middlebrook 7H11 selective agar with antimicrobials (Remel, Inc., Lexena, Kans.) and by using the fluorometric BACTEC technique (BACTEC MGIT 960 system; Becton-Dickinson Diagnostic Instrument Systems, Sparks, Md.) as previously described [11], [12]. Mycobacterial species were identified by biochemical testing [13].

Pulmonary function testing was performed by spirometry (MasterScreen, Jaeger, Germany or Vmax 6200, Sensormedics Corp., United States) according to the American Thoracic Society/European Respiratory Society guidelines[14]–[16]. Each machine was calibrated daily before the testing, which was done by trained technicians. Each patient performed at least three acceptable forced expiratory manoeuvres that fulfilled the criteria of repeatability [15].

### Data Collection

A standardized case record form was used to collect demographic and clinical data, including age, sex, body-mass index (BMI), history of smoking, chronic obstructive lung disease, asthma, prior history of pulmonary tuberculosis, radiographic findings, inhalation medication, and the course of anti-NTM treatment.

Chronic obstructive lung disease was diagnosed when the forced expiratory volume in 1 second (FEV_1_) and forced vital capacity (FVC) was <0.70. Pulmonary lesions were categorized into two predominant radiographic patterns, cavitary (fibro-cavitary) or bronchiectatic (nodular bronchiectasis), by chest radiography. Radiographic severity (RS) was recorded according to a previous study [17]. Briefly, each lung was divided into three areas and each area was rated on a four-point scale of 0 to 3 for extent of infiltration, with a maximum score of 18. The chest images were initially interpreted by two chest specialists (MR Lee and JY Wang) and in cases of discrepancy, the final recording was made after holding a discussion between them.

Inhalation medication was recorded if the patients had used long-acting muscarinic antagonist or long acting beta-2 agonist with/without inhaled corticosteroids.

### Statistical Analysis

Proportions or means were used to describe the demographic, clinical and radiographic characteristics. Inter-group differences were analyzed using independent-sampled *t* test for continuous variables and *chi*-square test for categorical variables. Linear regression analysis was used to identify factors associated with FEV_1_ and FVC decline on follow-up. In stepwise variable selection procedure, all of the potential predictors were included. Significance levels for entry and stay were set at 0.15. A two-sided *p*<0.05 was considered significant. All analyses were performed using the SPSS v13.0 (SPSS, Inc., Chicago, IL).

## Results

### Clinical Data

**Figure 1:**
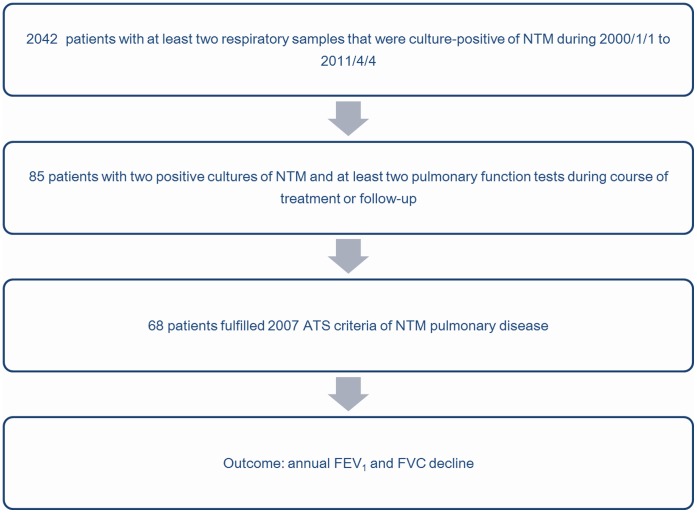
Selection of non-tuberculous mycobacterial pulmonary disease patients (n = 68) with at least two pulmonary function tests. **NOTE.** NTM, non-tuberculous mycobacteria; ATS, American Thoracic Society; FVC, forced vital capacity; FEV_1_, forced expiratory volume in 1 second.

In the patient selection process (Fig. 1), 2042 persons with at least two respiratory samples that were culture-positive for NTM were identified by searching the mycobacteria database. By further linking with the pulmonary function test database, 85 patients with at least two pulmonary function tests during the treatment course or follow-up were identified. Seventeen were excluded for lack of the diagnostic criteria of NTM pulmonary disease so 68 patients were included in the analysis.

Based on the demographic data (Table 1), the median age was 68 and 56 years for males and females, respectively, and there was male preponderance (62%). *Mycobacterium avium* complex were the most common NTM species isolated (53%), followed by *M. chelonae-abscessus* (34%). Of the 68 patients, 62% were either active smokers or ever-smokers. About one-third had prior history of tuberculosis. By radiographic pattern and score, the bronchiectatic (nodular bronchiectasis) pattern was common (72%) and 74% of patients had a radiographic score of ≤3.

**Table 1:** Clinical characteristics of patients with NTM pulmonary disease (n = 68).

Characteristic	Total(n = 68)	*MAC*(n = 36)	*M. che-abs*(n = 23)	*M. kansasii*(n = 9)
Age: median [range]	65 [30–88]	66.5 [30–88]	62 [46–85]	65 [52–82]
Male	41 (60%)	23 (64%)	10 (43%)	8 (89%)
Body-mass index: mean ± SD	20.6±3.8	20.5±3.9	20.1±3.5	21.8±4.0
<18.5	20 (29%)	9 (25%)	9 (39%)	2 (22%)
18.5–25	41 (60%)	23 (67%)	12 (52%)	6 (67%)
>25	7 (10%)	4 (11%)	2 (9%)	1 (11%)
Baseline PFT				
FEV_1_≥80%	24 (35%)	13 (36%)	10 (43%)	1 (11%)
50% ≤ FEV_1_<80%	21 (31%)	11 (31%)	7 (30%)	3 (33%)
30% ≤ FEV_1_<50%	19 (28%)	11 (31%)	4 (17%)	4 (44%)
FEV_1_<30%	4 (6%)	1 (3%)	2 (9%)	1 (11%)
Smoking status				
Active smoker	11 (16%)	6 (17%)	4 (17%)	1 (11%)
Ever smoker	31 (46%)	17 (47%)	8 (35%)	6 (67%)
Never smoker	26 (38%)	13 (36%)	11 (48%)	2 (22%)
Chronic obstructive pulmonary disease	34 (50%)	19 (53%)	8 (35%)	7 (78%)
Asthma	12 (18%)	6 (17%)	4 (17%)	2 (22%)
Past history of tuberculosis	23 (34%)	12 (33%)	9 (39%)	2 (22%)
Radiographic pattern				
Bronchiectasis	49 (72%)	25 (69%)	18 (78%)	6 (67%)
Cavitary	19 (28%)	11 (31%)	5 (22%)	3 (33%)
Radiographic score				
≤3	50 (74%)	25 (69%)	19 (82%)	6 (67%)
>3	18 (26%)	11 (31%)	4 (17%)	3 (33%)
Inhalation treatment	33 (49%)	22 (61%)	6 (26%)	5 (56%)
Ever receiving anti-NTM treatment	21 (31%)	12 (33%)	7 (30%)	2 (22%)
Mean interval of PFT (month)	47	47	47	48
Average annual FEV_1_ decline (ml/year)	48	19	72	104
Average annual FVC decline (ml/year)	91	81	60	205

**NOTE.** NTM, non-tuberculous mycobacteria; MAC, *Mycobacterium avium* complex; M. che-abs, *Mycobacterium chelonae-abscessus*; PFT, pulmonary function test.

Data are number (%) unless otherwise mentioned.

Inhalation medication was recorded if the patients had used long-acting muscarinic antagonist or long-acting beta-2 agonist with/without inhaled corticosteroids.

At baseline, 65% of all patients had impaired pulmonary function. The mean follow-up period of lung function testing was 47 months. The average FEV_1_ and FVC decline was 48 and 91 ml/year, respectively. On follow-up, 24 (35%) patients had no FEV_1_ decline and 35 (51%) had an FEV_1_ decline <30 ml/year.

By treatment regimens (Table 2), none of the MAC pulmonary disease patients received triple combination therapy, including macrolides, ethambutol and rifampicin, as suggested by the ATS guidelines [1]. Only one *M. chelonae-abscessus* patient received intravenous amikacin plus imipenem for 3 weeks, followed by clarithromycin monotherapy for 15 months. Two patients treated for *M. kansasii* pulmonary disease had regimens that were inadequate in number of drugs, dosage, and duration. None of the 68 patients received surgical intervention for NTM pulmonary disease.

**Table 2:** Anti-mycobacterial treatment regimens by NTM species.

NTM species	Regimen	Duration (month): median [range]	No. of patients
MAC	Macrolide monotherapy	6 (4–6)	4
	Quinolone monotherapy	7	1
	Macrolide plus quinolone	1 (0.25–12)	7
	Macrolide, ethambutol plus rifampicin		0
*M. chelonae-abscessus*	Macrolide plus quinolone	3.5 (0.5–16)	6
	Amikacin, imipenem, macrolide	15*	1
*M. kansasii*	Macrolide	0.3	1
	Macrolide plus quinolone	1	1

**NOTE.** NTM, non-tuberculous mycobacteria; MAC: *Mycobacterium avium* complex; M. che-abs: *Mycobacterium chelonae-abscessus.*

* This patient received parenteral amikacin and imipenem for 3 weeks, followed by oral clarithromycin for 14 months.

**Figure 2:**
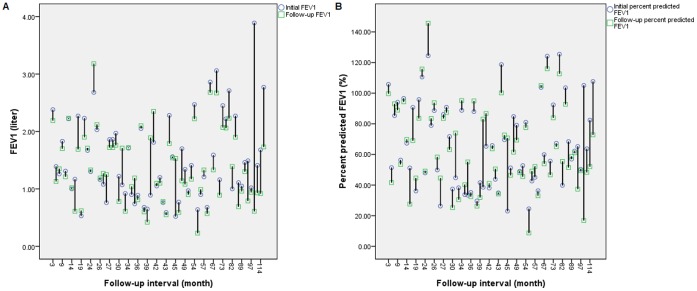
Illustration of initial and follow-up FEV_1_ (Figure 2A) and percent predicted FEV_1_ (Figure 2B) among 68 patients. Figure 2A. Initial and follow-up FEV_1_ Figure 2B. Initial and follow-up percent predicted FEV_1_ NOTE. FEV_1_, forced expiratory volume in 1 second.

In linear regression analysis, FEV_1_>50% (beta: 0.349, *p* = 0.002), male sex (beta: 0.295, *p* = 0.018), bronchiectatic pattern (beta: 0.232, *p* = 0.035), and radiographic score >3 (beta: 0.217, *p* = 0.049) were associated with greater FEV_1_ decline. Older age was associated with less FEV_1_ annual decline (beta: −0.472, *p* = 0.000) (Table 3). When using percent predicted FEV_1_ decline as outcome, younger age remains associated with greater decline in multivariate linear regression analysis (beta: 0.263, *p* = 0.035). A graph indicating initial and follow-up FEV_1_ and percent predicted FEV_1_ is demonstrated in Fig. 2 (2A and 2B).

**Table 3:** Factors associated with the amount of annual FEV_1_ decline in patients with NTM pulmonary disease, by linear regression analysis.

Variables	Beta	*p* value	95% C.I.
Age	−0.472	<0.001	−0.012–−0.004
Gender: male *vs*. female	0.295	0.018	0.022–0.229
Radiographic pattern: bronchiectasis *vs*. cavitary	0.232	0.035	0.008–0.205
Radiographic score: >3 *vs.* ≤3	0.217	0.049	0.000–0.202
Baseline function: FEV_1_>50% *vs*. FEV_1_≤50%	0.349	0.002	0.059–0.245

**NOTE.** NTM, non-tuberculous mycobacteria; FEV_1_, forced expiratory volume in 1 second.

With annual FVC decline as the outcome, only initial FEV_1_>50% of predicted value (beta: 0.263, *p* = 0.032) was significantly associated with greater FVC annual decline in linear regression. *M. kansasii* pulmonary disease was marginally associated with greater annual FVC decline (beta: 0.227, *p* = 0.062) (Table 4).

**Table 4:** Factors associated with the amount of annual FVC decline in patients with NTM pulmonary disease, by linear regression analysis.

Variables	Beta	*p* value	95% C.I.
*M. kansasii* pulmonary disease	0.227	0.062	−0.342–−0.008
Baseline pulmonary function: FEV_1_>50% *vs*. FEV_1_≤50%	0.263	0.032	0.012–0.263

**NOTE.** NTM, non-tuberculous mycobacteria; FVC, forced vital capacity; FEV_1_, forced expiratory volume in 1 second.

## Discussion

In this study, younger age, male sex, bronchiectatic pattern, higher initial radiographic score, and better baseline FEV_1_ are associated with greater FEV_1_ decline in patients with NTM pulmonary disease. Better baseline FEV_1_ is also associated with greater FVC decline. Clinicians should not neglect NTM pulmonary disease in patients who are young and have relatively preserved lung function. More extensive lung involvement, defined by higher radiographic score and a bronchiectatic pattern, can also help identify patients at greater risk of lung function decline.

The mean FEV_1_ decline among pulmonary NTM patients is 48 ml/year in the present study, significantly exceeding the normal annual FEV_1_ decline after adjustment for age (28.4–35.6 ml/year) [18] and greater than that observed in patients with chronic obstructive pulmonary disease (COPD) patients (42±1 ml/year) [19]. A large proportion (65%) of patients have abnormal baseline function test, which is consistent with a previous study [9]. Notably, not all patients have FEV_1_ decline during follow-up and more than half have an annual FEV1 decline <30 ml/year, which means that not all patients with NTM pulmonary disease have clinical deterioration during follow-up. This finding supports the NTM treatment guidelines, which state that the diagnosis of NTM pulmonary disease does not, per se, necessitate the institution of therapy, which is a decision based on potential risks and benefits of therapy for individual patients [1]. This highlights the importance of identifying NTM patients who are likely to have clinical deterioration as they may require prompt treatment [2].

A bronchiectatic pattern of NTM pulmonary disease has a greater annual FEV_1_ decline in the present study. Nodular bronchiectatic type, as coined by Wallace et al., refers to centri-lobular nodular lesions associated with bronchiectasis [20]. Nodular bronchiectatic NTM pulmonary disease pattern is currently considered secondary to pre-existing bronchiectasis that predisposes to NTM infection and disease [21]. Bronchiectasis patients have a natural course of gradual worsening of symptoms and decline of lung function, primarily FEV_1_ [22]. It is therefore not surprising that nodular bronchiectatic pattern is a risk factor for greater FEV_1_ decline. In analyzing FVC decline, bronchiectasis is not significantly associated with lung function decline, which is consistent with a prior study wherein the degree of FVC decline is less than the FEV_1_ decline (−1.2% vs. −3.8%, with median follow-up of 28 months) in bronchiectasis patients with serial follow-up [22].

The finding that male sex is associated with greater FEV_1_ decline is likewise compatible with a previous study [23]. However, in contrast to literature reports showing that FEV_1_ decline accelerates with aging [24], [25], the present study shows that younger age predicts greater FEV_1_ decline. Concerns exist with the finding that older age is associated with lower FEV_1_ decline because if baseline FEV_1_ is lowered with age, there is less scope for a reduction. By using percent predicted FEV_1_ decline as outcome, we find that younger age remains associated with greater decline in multivariate linear regression analysis.

In this study, the finding that better baseline lung function predicts greater FEV_1_ decline is unexpected. Earlier studies reveal that smokers with lower FEV_1_ levels have a steeper FEV_1_ decline, which is known as horse-racing effect [26]. However, recent studies targeting COPD patients reveal just the opposite: patients with milder lung function impairment have quicker FEV_1_ decline than patients with more severely impaired lung function [27]. Initial lung function may be particularly important because initial FEV_1_ is the only factor involved in both FEV_1_ and FVC decline. Findings in the present study suggest that young men with good lung reserve may decelerate to a greater extent during NTM infection. It is noteworthy, however, that this group of patients may look healthy and anti-NTM therapy may thus be considered unnecessary for them.

*M. kansasii* pulmonary disease is borderline associated with annual FVC decline in this study. The rate of both FEV_1_ and FVC decline (104 and 205 ml/year, respectively) is higher in patients with *M. kansasii* pulmonary disease compared with the other two species. Untreated *M. kansasii* pulmonary disease leads to clinical symptoms and has a progressively deteriorating course [28]. Another explanation may be the lack of treatment in these patients. Due to the limited case number and borderline significance, it cannot be concluded that patients with *M. kansasii* pulmonary disease have greater annual FVC decline.

In this study, NTM pulmonary disease was diagnosed in 68 out of 85 patients with at least two NTM clinical isolates from respiratory specimens. Compared to a previous study, the proportion of patients with NTM disease is higher (80% *vs.* 55%) [2]. This is probably because patients who had undergone at least two pulmonary function tests are likely to have more symptoms and more deterioration during follow-up, and are therefore likely to have NTM pulmonary disease rather than mere colonization.

The present study has several limitations. First, its retrospective nature makes it impossible to include all NTM pulmonary disease patients with a standard protocol of follow-up. There is no standardized anti-NTM treatment or quantified use of aerosol airway medications. However, the latter should not be a serious concern since there is no solid evidence showing that these can alter the rate of FEV_1_ decline [19]. Second, biochemical method rather than the preferred molecular method was used to identify NTM species. Detailed species differentiation between MAC and *M. chelonae-abscessus* was also not performed. Though epidemiologically and therapeutically significant, the molecular method is not readily available in many laboratories and may not be practical in clinical practice [29], [30]. Third, this study does not have a control group (for example, patients with pulmonary tuberculosis and patients with other pulmonary diseases).

In conclusion, factors influencing the rate of lung function decline among patients with NTM pulmonary disease are younger age, male sex, better baseline lung function, bronchiectatic radiologic pattern, and higher radiographic score. These are associated with rapid decline of FEV_1_. A more aggressive treatment approach should be considered in NTM pulmonary disease patients with these risk factors.
